# Clinical Oversight and Delayed Diagnosis of a Pathological Compression Fracture Causing Paraplegia

**DOI:** 10.7759/cureus.68296

**Published:** 2024-08-31

**Authors:** Yin-Sheng Chen, Ping-Chuan Liu, Chih-Chang Chang, Tsung-Hsi Tu, Chao-Hung Kuo

**Affiliations:** 1 Department of Neurosurgery, Taipei Veterans General Hospital, Taipei, TWN; 2 School of Medicine, National Yang Ming Chiao Tung University, Taipei, TWN; 3 Department of Biomedical Engineering, School of Biomedical Science and Engineering, National Yang Ming Chiao Tung University, Taipei, TWN; 4 Ph.D. Program in Medical Neuroscience, College of Medical Science and Technology, Taipei Medical University, New Taipei City, TWN

**Keywords:** biopsy, spine surgery, pathological compression fracture, metastatic spinal tumors, extradural compression

## Abstract

While osteoporosis is the primary cause of vertebral compression fractures (VCFs), it's crucial to promptly recognize pathological fractures through comprehensive diagnostic tests, including vertebral biopsies, to determine the exact etiology. For instance, a 66-year-old male with osteoporosis experienced worsening lower limb weakness and back pain after an initial vertebroplasty for a T12 compression fracture. Subsequent MRI revealed severe circumferential extradural compression at T12, leading to further surgeries that eventually uncovered metastatic adenocarcinoma from a pancreatic tumor. This case highlights the importance of precise diagnosis through vertebral biopsy and the necessity of sufficient ventral decompression or corpectomy, coupled with extensive laminectomy, to address severe neurological impairments like paraplegia. Prompt and accurate interventions can significantly improve patient outcomes and quality of life.

## Introduction

Vertebral compression fractures (VCFs), characterized by severe pain and spinal deformities, severely limit a patient's ability to perform daily activities, often leading to immobility, psychological distress, and heightened morbidity and mortality. The primary causes of VCFs are osteoporosis and metastatic disease. Osteoporotic VCFs occur in approximately 30-50% of individuals over the age of 50 with osteoporosis and in about 12% of those aged 50-79 globally, affecting an estimated 1.4 million patients annually [1,2]. VCFs resulting from spinal metastases vary depending on the primary cancer. A systematic review of 56 studies found that 12.63% of patients with spinal metastases experience pathological VCFs [3], which often result from direct osteolysis by tumor cells, bone loss due to radiation therapy, hormone or steroid use, and overall poor health [4]. Rapid progression of these fractures can cause severe pain, instability, and neurological deficits, including paraplegia. Early diagnosis and treatment are crucial for improving outcomes, prognosis, and quality of life for affected patients. The clinical features and diagnosis of malignant VCFs are well documented, with key red flags including a history of malignancy, older age, weight loss, and persistent back pain [5]. In one reported case, a 78-year-old man initially treated for an osteoporotic fracture at L3 later developed symptoms suggestive of malignancy. Magnetic resonance imaging (MRI) findings showed significant changes, and a biopsy confirmed metastatic squamous cell carcinoma [6]. Despite the presence of clinical indicators, early diagnosis of malignant VCFs remains challenging, as these fractures are often misdiagnosed as benign osteoporotic fractures, even after a thorough medical history and comprehensive examinations. Current guidelines lack standardized diagnostic recommendations for effectively managing such cases. Through this case study, we aim to present strategies for enhancing early detection and improving diagnostic accuracy.

## Case presentation

**Figure 1:**
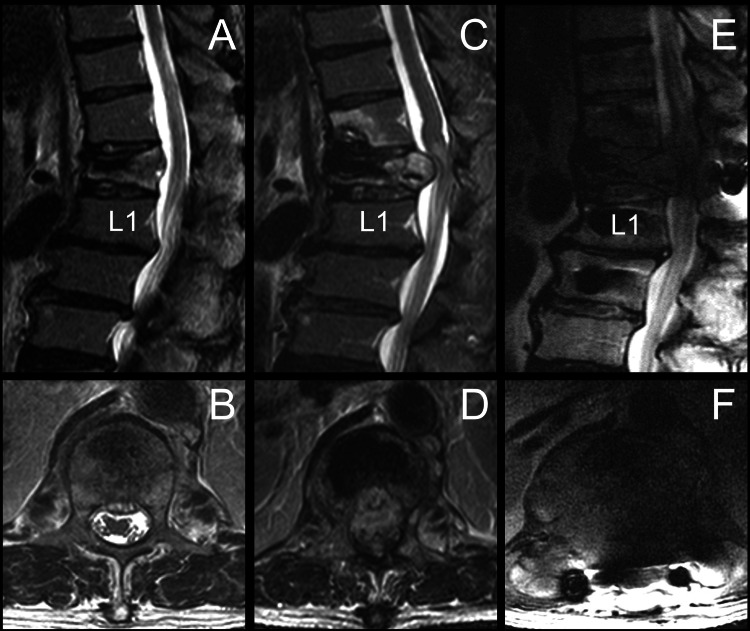
MRI series depicting a T12 compression fracture The MRI series depicting a T12 compression fracture is presented as follows: The initial T2-weighted images ((A) sagittal view; (B) axial view) reveal a T12 compression fracture with heterogeneous signals and no significant extradural compression. Six months following T12 vertebroplasty, the sagittal (C) and axial (D) images show a further reduction in vertebral height and anterior bulging, leading to extradural compression. The patient subsequently underwent a T12 laminectomy with fixation from T10 to L2. However, three months after this secondary surgery, the patient experienced a rapid neurological decline. Follow-up MR images in sagittal (E) and axial (F) views demonstrate progressive extradural compression of the spinal cord. MRI: magnetic resonance imaging

A 66-year-old man experienced lower back pain following a fall. Initial neurological assessments showed normal muscle strength with no urinary or fecal dysfunction. Imaging of the lumbar spine revealed a compression fracture at the T12 vertebra, while an MRI indicated heterogeneous signals without significant extradural compression (Figure 1A, 1B).

**Figure 2:**
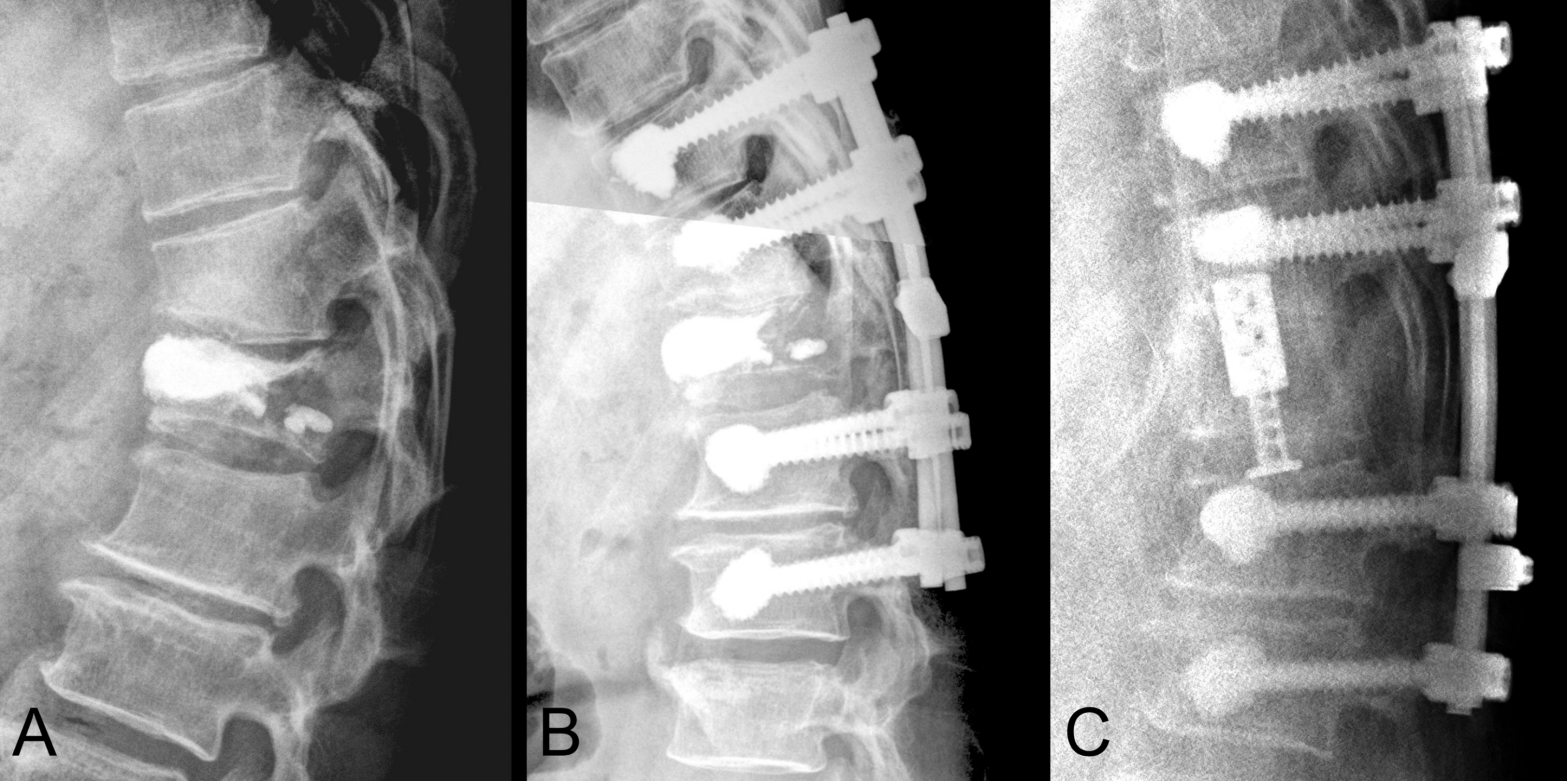
X-ray series of a T12 compression fracture (A) Sagittal view of the lumbar spine radiograph after T12 vertebroplasty. (B) Sagittal view of the lumbar spine radiograph after T12 laminectomy and fixation from T10 to L2. (C) Sagittal view of the lumbar spine radiograph after T12 partial corpectomy, expandable prosthesis reconstruction, and fixation.

A bone mineral density (BMD) test conducted before surgery, using dual-energy X-ray absorptiometry (DEXA), showed a T-score of -2.7. The patient underwent T12 percutaneous vertebroplasty (Figure 2A) initially alleviating symptoms.

**Figure 3:**
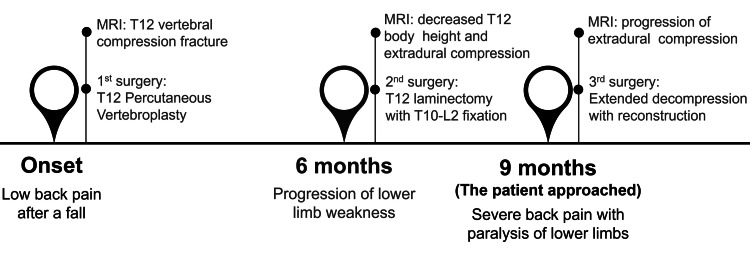
Summary of the clinical history organized according to the timeline

However, progressive bilateral lower limb weakness (graded as 3/5) developed over time. A follow-up MRI at six months showed T12 vertebral with more decreased vertebral height and ventral enlargement, resulting in extradural compression (Figure 1C, 1D). Subsequently, the patient underwent T12 total laminectomy with T10 to L2 fixation (Figure 2B). Three months post-surgery, he presented with exacerbated symptoms, including severe back pain and near-total paralysis of the lower limbs. Neurological examination revealed severe weakness (graded as 1/5) in the lower extremities with intact sensory function. Due to the rapid deterioration of neurological function, the patient was transferred to our hospital. MRI indicated progressive extradural compression (Figure 1E, 1F). Extensive total laminectomies were performed, along with T12 partial corpectomy and expandable prosthesis reconstruction and fixation (Figure 2C). Intraoperative findings suggested severe bony destruction indicative of malignancy. The clinical history is summarized in Figure 3.

**Figure 4:**
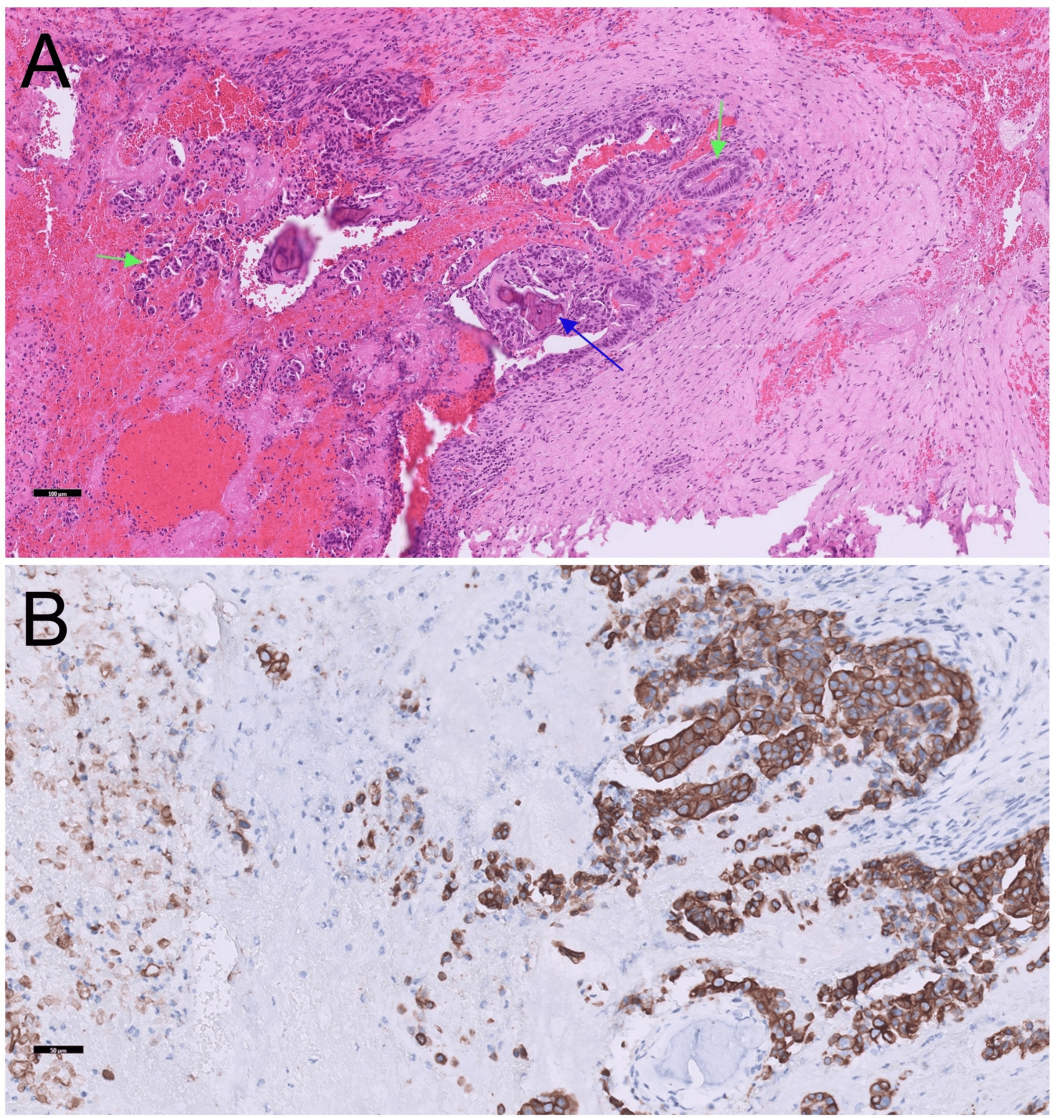
Histopathological presentations of T12 epidural lesion H&E staining of the extradural mass revealed bone (indicated by the blue arrow) and metastatic adenocarcinoma (indicated by the green arrows) ((A) H&E stain, scale bar=100 μm, 10× magnification). Immunostaining for CK7 ((B) scale bar=50 μm, 20× magnification) and CDX-2 was positive in the tumor cells. These findings are consistent with metastatic adenocarcinoma, likely originating from the gastrointestinal or pancreatobiliary tract. H&E: hematoxylin and eosin; CK7: cytokeratin 7; CDX-2: caudal-type homeobox 2

Upon further pathological examination, tumor markers were scrutinized, revealing an elevation in carbohydrate antigen 19-9 (CA 19-9) levels (2334 U/mL). Histopathological analysis of the T12 vertebral body and its adjacent soft tissues unveiled infiltration by tumor cells exhibiting an irregular glandular pattern. Noteworthy findings included extensive hemorrhage and pronounced tumor necrosis (Figure 4A). The tumor cells exhibited positivity for cytokeratin 7 (CK7) and caudal type homeobox 2 (CDX-2) but negativity for cytokeratin 20 (CK20), paired box 8 (PAX8), NK3 homeobox 1 (NKX3.1), and thyroid transcription factor 1 (TTF-1) (Figure 4B).

These features align with those of metastatic adenocarcinoma, potentially originating from the gastrointestinal tract, pancreatobiliary tract, or other sources. MR cholangiopancreatography was performed, revealing focal dilatation of the common bile duct without significant intraductal lesions. The imaging results suggested the presence of a pancreatic head or ampulla of Vater tumor. Subsequently, the patient initiated chemotherapy. Postoperatively, back pain improved significantly, but paralysis showed minimal improvement, necessitating continued rehabilitation.

## Discussion

Differentiating between osteoporotic and pathological compression fractures early on is challenging. While clinical history and laboratory tests, such as tumor markers, can raise suspicion for malignant conditions, most literature emphasizes imaging features. Different modalities on MRI can help distinguish between osteoporotic and malignant VCFs [7]. Benign VCFs typically show normal posterior element signal [8], retropulsed bone fragments [9,10], and additional benign VCFs throughout the spine [11]. In contrast, malignant VCFs often present with abnormal pedicles or posterior element signal [9,11,12], epidural or paravertebral soft tissue mass [9,13,14], expanded posterior vertebral contour [9,11], or metastasis in other vertebrae [9,10]. Regarding signal intensity and enhancement patterns, osteoporotic VCFs may retain normal marrow signals with high T1 and intermediate T2, have well-defined margins, or show a "fluid sign" (a focal, linear, or triangular area of T2 hyperintensity [9]). Conversely, malignant VCFs often exhibit geographic replacement of normal marrow signal with diffuse homogeneous low signal intensity and irregular margins. On diffusion-weighted imaging, diffusion is generally increased in osteoporotic fractures due to bone marrow edema, while it is typically restricted in malignant VCFs because of the high cellularity of tumor tissue [15].

A vertebral biopsy is an option for verifying the pathological process and confirming the diagnosis of metastatic spinal fractures. In previous literature, there is no consensus regarding the necessity of performing a vertebral biopsy before or during surgical treatments such as vertebroplasty in VCFs. Several studies have addressed this issue, with most supporting the utility of the biopsy, considering it to lead to minimal increases in morbidity and operation time [16,17]. A retrospective analysis of vertebral biopsies performed on 234 patients excluded those with a history of neoplasm or imaging suspicious for pathological fractures [18]. The results showed that 6.2% of biopsies demonstrated unexpected histological positivity, including multiple myeloma, metastasis, and lymphoma/leukemia, with only 2.8% of biopsies remaining undiagnostic. The conclusion was that a vertebral collapse should always be investigated with a biopsy before performing the consolidation procedure. Thus, for VCFs, it is suggested that vertebral biopsy should be routinely performed, especially in cases with rapid progression of symptoms, highly suspicious findings on imaging, and a history of cancer.

Various management strategies can be employed in cases of tumor-related spinal fractures, with surgical interventions often dictated by several significant factors. These factors include the degree of epidural cord compression, radiosensitivity of the tumor, presence of spinal instability, patient medical status, and estimated survivorship [19]. Surgical options include decompression with stabilization, en bloc resection, percutaneous kyphoplasty/vertebroplasty, or minimally invasive corpectomy [19]. When epidural spinal cord compression is present, decompression with stabilization is commonly recommended after a thorough evaluation of the patients' general medical condition, performance status, life expectancy, and personal preferences. Current literature provides limited information regarding specific surgical intervention methods for metastatic spinal fractures leading to extradural compression. In cases of severe spinal stenosis, an extensive laminectomy and fixation spanning the diseased segments is suggested. For sufficient ventral side decompression, a corpectomy with stabilization and support of the anterior column may be indicated.

## Conclusions

Early identification and diagnosis of pathological VCFs are challenging but crucial for better treatment outcomes and prognosis. Imaging features indicative of malignancy and vertebral biopsy, a safe and effective procedure, are essential for accurate diagnosis and management. Routine vertebral biopsies before treatment are recommended, especially in rapidly progressing compression fractures, to avoid diagnostic delays and ensure timely intervention. Additionally, rapidly progressing metastatic spinal compression fractures may cause severe neurological impairments due to extradural compression. Adequate ventral decompression or corpectomy, along with extensive laminectomy, is necessary in such situations.
